# Dysregulation of the Lateral Habenula in Major Depressive Disorder

**DOI:** 10.3389/fnsyn.2018.00046

**Published:** 2018-12-07

**Authors:** Caroline A. Browne, Robert Hammack, Irwin Lucki

**Affiliations:** Department of Pharmacology and Molecular Therapeutics, Uniformed Services University of the Health Sciences, Bethesda, MD, United States

**Keywords:** lateral habenula, major depressive disorder, behavior, optogenetic, reward, aversion

## Abstract

Clinical and preclinical evidence implicates hyperexcitability of the lateral habenula (LHb) in the development of psychiatric disorders including major depressive disorder (MDD). This discrete epithalamic nucleus acts as a relay hub linking forebrain limbic structures with midbrain aminergic centers. Central to reward processing, learning and goal directed behavior, the LHb has emerged as a critical regulator of the behaviors that are impaired in depression. Stress-induced activation of the LHb produces depressive- and anxiety-like behaviors, anhedonia and aversion in preclinical studies. Moreover, deep brain stimulation of the LHb in humans has been shown to alleviate chronic unremitting depression in treatment resistant depression. The diverse neurochemical processes arising in the LHb that underscore the emergence and treatment of MDD are considered in this review, including recent optogenetic studies that probe the anatomical connections of the LHb.

## Introduction

Distinct physiological and molecular activity in a small region located in the most caudal and distal part of the epithalamus, known as the habenula, regulates circuit based alterations associated with the progression of depression in humans (Sartorius et al., 2010) and depressive-like behavior in rodents (Shabel et al., 2014). Consisting of two anatomically and functionally discrete nuclei, the lateral (LHb) and medial habenula (MHb) inhibit the monoaminergic centers of the midbrain (Namboodiri et al., 2016). Until recently, the heterogeneous pathophysiology of major depressive disorder (MDD) has hindered the development of rapid-acting treatments for the millions of people suffering worldwide from this debilitating and lifelong illness (Major Depressive Disorder Working Group of the Psychiatric et al., 2013). However, accumulating evidence suggests that conventional and novel antidepressant therapies target LHb functions to remediate depressive symptoms. This review will highlight diverse neurochemical and molecular processes arising in the LHb that underscore the emergence and treatment of MDD. Additionally, we will summarize the findings of recent optogenetic studies probing the anatomical connections that facilitate the complex behavioral processes regulated by the LHb.

### LHb Inputs and Outputs

**FIGURE 1:**
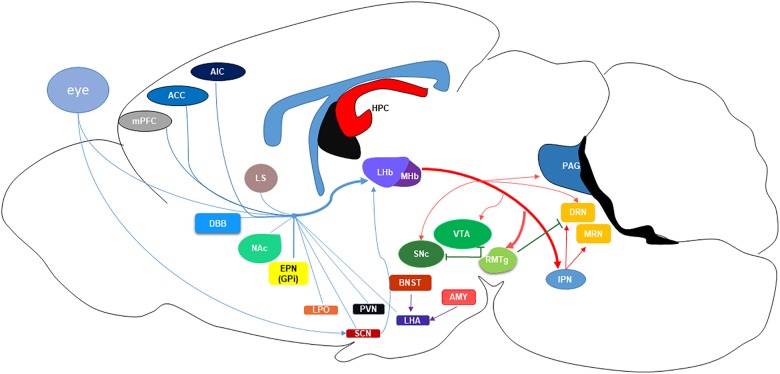
Schematic of the projections to and from the lateral habenula (LHb) nucleus. MHb, medial habenula; mPFC, medial prefrontal cortex; ACC, anterior cingulate cortex; AIC, anterior insular cortex; LS, lateral septum; NAc, nucleus accumbens; DBB, diagonal band of Broca; EPN (GPi), entopeduncular nucleus (globus pallidus); LPO, lateral preoptic area; PVN, paraventricular nucleus; SCN, suprachiasmatic nucleus; LHA, lateral hypothalamic area; BNST, basal nucleus of the stria terminalis; AMYG, amygdala; HPC, hippocampus; SNc, substantia nigra pars compacta; VTA, ventral tegmental area; RMTg, rostromedial tegmental nucleus; IPN, interpeduncular nucleus; PAG, periaqueductal gray; DRN, dorsal raphe nucleus; MRN, median raphe nucleus.

Both LHb and MHb receive inputs through the stria medullaris (SM) and send their outputs through the fasciculus retroflexus (FR) (Namboodiri et al., 2016). Together these four structures create the dorsal diencephalic route, connecting limbic forebrain structures to the midbrain (Sutherland, 1982). The ability of the habenular nuclei to control dopamine (DA) and serotonin (5-HT) is evolutionarily conserved across species (Stephenson-Jones et al., 2012). On the ventral side of the MHb, cholinergic neurons account for two-thirds of the neurons, with the dorsal third expressing substance P positive neurons. A common feature of most neurons in the MHb is co-release of glutamate (Namboodiri et al., 2016). The MHb receives input from the medial and lateral septum and projects mainly to the interpeduncular nucleus (IPN) (Hikosaka, 2010). In contrast, the LHb is primarily a glutamatergic nucleus, although some GABAergic interneurons have also been found in this region (Smith et al., 1987; Lecca et al., 2014). The ability of the LHb to control monoamine neurotransmission underpins the initial attraction of this region in the context of MDD, as the monoamine hypothesis of depression dictates that diminished or depleted monoamine neurotransmission is the primary causal factor in precipitating a depressive episode, or impairing recovery/responsiveness to antidepressant treatment (Heninger et al., 1996). As depicted in Figure 1. the primary inputs to the LHb are the globus pallidus (GPi), or non-primate equivalent entopeduncular nucleus (EPN), lateral hypothalamic area (LHA), paraventricular nucleus (PVN), lateral preoptic area (LPO), ventral pallidum (VP), and the basal forebrain (BF) consisting of the lateral septum (LS), nucleus accumbens (NAc), and diagonal band nuclei (DBN) (Lecca et al., 2014). Projections from the dorsal and pregenual anterior cingulate cortex (ACC), anterior insula cortex (AIC), and caudolateral orbitofrontal cortex (OFC) to the LHb were defined using diffusion tensor imaging in humans (Vadovicova, 2014), confirming earlier electron microscopy and biotinylated dextran amine labeling studies in rats (Greatrex and Phillipson, 1982; Kim and Lee, 2012). LHb efferents robustly inhibit the aminergic nuclei of the midbrain, dopamine (DA) in the ventral tegmental area (VTA) and substantia nigra pars compacta (SNc) (Christoph et al., 1986; Namboodiri et al., 2016), and serotonin (5-HT) in the dorsal and median raphe nucleus (DRN and MRN) (Wang and Aghajanian, 1977; Namboodiri et al., 2016).

Thus, the LHb acts as a relay center for cortical and limbic control of global monoaminergic neurotransmission. LHb projections locally inhibit DA neurons and GABAergic interneurons within the VTA at equal rates, approximately 45% and 52%, respectively (Omelchenko et al., 2009; Goncalves et al., 2012; Brown and Shepard, 2016). In the DRN, LHb GABA and glutamatergic terminals regulate 5-HT cell bodies, however, DRN GABA interneurons primarily receive glutamatergic projections from the LHb (Zhou et al., 2017). These local GABAergic interneurons in the DRN are critical regulators of 5-HT release and behavior (Challis et al., 2013). Moreover, the ability of LHb projections to stimulate GABA neurons are demonstrably greater than other long-range inputs to the DRN (Zhou et al., 2017). In addition to the local inhibition of monoamines, the LHb primarily regulates DA and 5-HT release through rostromedial tegmental (RMTg) projections. The majority of LHb glutamatergic projections activate the GABAergic RMTg, also known as the tail of the VTA, which in turn robustly inhibits monoamine release from midbrain nuclei (Stamatakis and Stuber, 2012; Stuber et al., 2012; Sego et al., 2014; Zhao et al., 2015). Therefore, the LHb is positioned to directly and indirectly inhibit release of monoamines and consequently offset the ability of conventional antidepressant therapies to alleviate depressive symptoms through elevation of monoamine concentrations in cortical and mesolimbic areas. This is important because these regions are known to be dysregulated in MDD (Drevets et al., 2008).

### Functions of the LHb

The LHb mediates a diverse range of behavioral responses. This section will briefly outline the primary behaviors influenced by LHb modulation. This includes its role in aversion, goal-directed processing, behavioral flexibility and social behavior, all behavioral domains that are dysregulated in MDD.

#### Reward and Aversion

Appropriate allocation of sensory processing in a real time manner is necessary to detect the salience of a stimulus, so that an individual can react to maximize reward for effort expended (Proulx et al., 2014). Reward processing is known to differ between healthy controls and depressed subjects (Treadway and Zald, 2011; Fletcher et al., 2015; Liu et al., 2017; Kumar et al., 2018), however, it is unknown whether one, or more of the subcomponents of reward processing is sufficient to drive negative affect and anhedonia. This includes a motivational component, incentive salience or “wanting, the hedonic quality of a reward, and the implicit and explicit associations an individual has formed with the stimulus” (Berridge and Kringelbach, 2008).

Although diverse nuclei help regulate reward processing, incentive salience is generally attributed to the VTA (Proulx et al., 2014) and its DAergic projections (Bromberg-Martin et al., 2010; Alcaro and Panksepp, 2011; Soskin et al., 2013; Fletcher et al., 2015; Olney et al., 2018), which are tightly controlled by glutamatergic LHb and GABAergic RMTg projections. Elegant reward-biased visual saccade studies conducted in non-human primates, demonstrated that LHb neurons regulate DA driven reward-prediction responses. Briefly, LHb firing increased in the presence of a stimulus associated with no reward and following omission of a predicted reward (Matsumoto and Hikosaka, 2007), and under these same conditions phasic firing of SNc DAergic neurons decreased. Conversely, firing of the LHb was inhibited in response to a stimulus predictive of reward and upon presentation of reward, and simultaneously SNc DAergic activity increased (Matsumoto and Hikosaka, 2007). Importantly, this study demonstrated that activation of the LHb was sufficient to suppress DA phasic firing in response to a reward, illustrating the powerful capacity of the LHb to inhibit DA responding (Matsumoto and Hikosaka, 2007).

In the non-human primate it has also been shown that the LHb is highly sensitive to the motivational valence of the conditioned stimulus, where the LHb exhibited increased activity in response to stimuli associated with negative outcomes (Matsumoto and Hikosaka, 2009a). These findings support the consistent observation that activation of the LHb drives conditioned place aversion in rodents (Friedman et al., 2011; Lammel et al., 2012; Stamatakis and Stuber, 2012). This is particularly relevant for psychiatric conditions such as post-traumatic stress disorder (PTSD), which is frequently comorbid with MDD (Hammack et al., 2012) and one that shares an overlapping neurobiology with MDD. The DRN and limbic structures regulated directly and indirectly by the LHb are critical in the acquisition of aversive associations required to produce learned helplessness (LH) and conditioned defeat (Hammack et al., 2012). Indeed, elevated metabolic activity was evident in the LHb during acquisition and recall of conditioned fear (Gonzalez-Pardo et al., 2012). Moreover, the intensity of the aversive association is also encoded by the LHb. Inhibitory avoidance to a weak shock stimulus (0.4 mA) persists for up to 48 h, but step-down inhibitory avoidance to a slightly stronger shock stimulus (0.8 mA) can persist for more than 1 week (Tomaiuolo et al., 2014). Inactivation of the LHb by muscimol microinjection 30 min prior to training, blocked inhibitory avoidance in the step down task 1 week post shock, suggesting that the LHb is involved in the latent reconsolidation and recall of aversive associations (Tomaiuolo et al., 2014). These findings agree with those in non-human primates, which indicated that the LHb exhibits context dependent neuronal firing (Matsumoto and Hikosaka, 2009b). The functional LHb connections that regulate reward and aversion will be discussed further in section “LHb Circuits”.

As illustrated in this section, the LHb has a key role in modulating behavioral domains in rodents and non-human primates relating to reward processing. Recent data has extended these findings to humans, where the greater the number of depressive episodes experienced by an individual was associated with increasingly blunted reward prediction error, which was positively correlated to increased punishment predictive error (bias toward negative outcomes) encoded by the LHb (Kumar et al., 2018). As such, future studies examining the impact of the LHb on depressive phenotypes should continue to place emphasis on the role of the LHb in negative outcome biases.

#### Goal-Directed Processing and Behavioral Flexibility

Blunted hedonic responding and impaired goal-directed cognitive processes are highly intertwined and consistently demonstrated in depressed patients (Treadway and Zald, 2011; Roiser and Sahakian, 2013; Soskin et al., 2013; Fletcher et al., 2015). Indeed, as many as 50% of MDD patients report deficits in memory, attention, executive function and processing speed despite antidepressant treatment (Culpepper et al., 2017; Kaser et al., 2017).

The LHb is necessary for monitoring the environment for “guide choices,” signals that enable the individual to switch behaviors to obtain rewards, or avoid punishment (Stopper and Floresco, 2014; Baker et al., 2015). As a relay hub, the LHb processes information from EPN efferents involved in emotional and motivational processing, from LHA and LPO efferents that are essential for sustained attention and arousal (Mizumori and Baker, 2017), and from cortical inputs which facilitate top-down regulation of behavior (Kim and Lee, 2012; Mathis and Lecourtier, 2017). These regions are central to the execution of complex cognitive tasks and their efferents to the LHb modulate midbrain aminergic release to facilitate behavioral flexibility (Baker et al., 2015; Baker and Mizumori, 2017). Specifically, DA is required for signal tracking and prediction of outcomes particularly in aversive states (Bromberg-Martin et al., 2010). The LHb maintains tonic inhibitory tone of DA release in the cortex, NAc and striatum, regions that are central to the induction or suppression of behavioral responses. LHb inactivation by administration of AMPA/kainate antagonists evoked transient increases in DA release in the NAc and striatum by approximately 60%. LHb inactivation also produced a 35% increase from baseline in PFC extracellular DA release. However, extracellular release of DA in the PFC was elevated for a considerably shorter period of time, than that observed in the NAc and striatum (Lecourtier et al., 2008). Conversely, VTA stimulation resulted in a pronounced increase in PFC DA release (∼150%) (Lecourtier et al., 2008). Given that LHb activation suppresses VTA activity, this data illustrates the powerful control of the LHb on VTA dopamine release in VTA projections sites. It has subsequently been established that the DA neurons stimulated by LHb efferents in the medial posterior VTA send ∼80% of their projections to the mPFC, whereas less than 10% of this VTA DA population project to the NAc (Lammel et al., 2012). This data emphasizes the importance of LHb control of VTA-PFC neurotransmission particularly in relation to behaviors influenced by PFC DA release. Similarly, 5-HT has been implicated in associative learning and decision making, which is enhanced with antidepressant compounds (Robbins, 2002; Bari and Robbins, 2013). Additionally, reduction in LHb norepinephrine release has been shown to reduce arousal (Purvis et al., 2018), which is required for sustained engagement in cognitive tasks.

The importance of the LHb in cognition and behavioral flexibility was demonstrated by lesion studies, LHb inactivation resulted in impaired performance in a 5-choice serial reaction time task (5-CSRTT) in rats (Lecourtier and Kelly, 2005). This finding supports the hypothesis that the LHb is critical for developing choice biases during evaluation and agrees with data obtained from delayed discounting tasks, where LHb inactivation in rats abolished probabilistic discounting for greater rewards, resulting in indiscriminate lever pressing for stimuli of lesser, or greater value (Stopper and Floresco, 2014). In line with these data, a less reactive LHb [illustrated by less c-Fos and brain derived neurotrophic factor (BDNF) immunoreactivity] in response to unpredictable chronic mild stress (CMS) exposure was evident in male and female Long-Evans rats that had extensive effort-based reward training in a foraging task. Despite stress exposure these animals exhibited greater perseveration of problem solving during task engagement relative to untrained animals (Kent et al., 2018). Overall this suggests that the LHb may be of great importance in the development of stress resilience.

Cortical LHb inputs are critical to the emergence and maintenance of behavioral flexibility. The lateral two-thirds of the LHb are innervated by ACC projections (Kim and Lee, 2012). Reversal learning in monkeys has revealed that ACC neurons store outcome information from previous experiences and direct subsequent behavioral adjustments. In contrast LHb neurons react more quickly to the stimulus than ACC neurons, and store negative outcomes relevant to the ongoing behavior and do not drive subsequent behavioral shifts (Kawai et al., 2015). Projections from the mPFC directly innervate the medial portion of the LHb (Greatrex and Phillipson, 1982; Kim and Lee, 2012) and are essential for relaying top-down information about environmental stimuli required to guide behavioral performance in tasks that demand high rates of sustained attention and engagement. Rats trained to respond for sucrose pellets in an operant delayed non-matching to position (DNMTP) paradigm exhibited deficits in working memory following muscimol inactivation of the LHb (Mathis et al., 2017). Additionally, mPFC/prelimbic-LHb disconnection, mimicked the effects of LHb silencing, ultimately reducing the temporal capacity to store relevant information required to perform this working memory task (Mathis et al., 2017). Thus, alterations in the activity of mPFC or LHb can impair performance in reward responding and behavioral flexibility.

Activation of the LHb is repeatedly demonstrated in working memory tasks. Faster acquisition, time to find the hidden platform in a Morris water maze task, in young rats correlated with greater regional cytochrome oxidase (CO) activity in the LHb when compared to aged rats, which exhibited less CO activity in the LHb and slower acquisition times in the Morris water maze (Villarreal et al., 2002). Additionally, rats that learned to discriminate odors for reinforcement showed increased activity in the LHb following a retrieval test (Tronel and Sara, 2002), illustrating the importance of the LHb in reward learning. The LHb is most active during acquisition, as lesioning of the LHb prior to training resulted in more errors in the Morris water maze task than sham lesioned rats (Thornton and Davies, 1991). In contrast, LHb lesioning following acquisition did not affect the performance of animals in the water maze once they had learned the location of the platform (Thornton and Davies, 1991). This finding was replicated by other labs, which demonstrated that lesioning of the LHb impaired performance (acquisition) in a Morris water maze task relative to sham controls (Lecourtier et al., 2004). Similarly, lesions of the LHb in hemiparkinsonian rats produced improvements in T-maze reward alternation task, a working memory task that requires rats to respond for rewards and resulted in elevated DA and 5-HT release in the PFC (Du et al., 2018). Although no direct anatomical connection between the dorsal HPC and the LHb has been identified, the ability of these regions to coordinate spatial memory is potentially mediated through LHb excitation/inhibition of 5-HT raphe-hippocampal projections (Zagami et al., 1995). More recently it has been suggested that LHb and dHPC are functionally coupled through theta oscillatory activity, which is driven by basal forebrain and VP inputs to the LHb (Goutagny et al., 2013). Together, these data illustrate the importance of the LHb in acquisition and recall of learned cue discriminators required to perform working memory tasks.

Overall, these data highlight a dynamic orchestration of cortical-LHb circuits during complex cognitive tasks, that are known to be significantly blunted in human MDD subjects. Although the extent to which the LHb influences these cortical regions is unknown, there is a growing interest in exploring the facets of cognition that are regulated by the LHb in the context of MDD and other stress-related illness.

#### Social Behavior

In addition to the regulation of goal-directed processing and working memory, mPFC-LHb projections regulate a wide range of social behaviors. Previously, it has been shown that increased excitation/inhibition imbalance in the cortex driven optogenetically by step function opsins which depolarize neurons for long periods of time [adeno-associated virus (AAV) expressing SSFO–EYFP with CaMKIIα promotor for excitation] reduced social exploration after SSFO activation in the mPFC but not in the primary visual cortex (Yizhar et al., 2011). Following on from this work, the LHb has now been identified as a critical PFC projection site in the regulation of social behavior (Benekareddy et al., 2018). Chemogenetic activation of mPFC neurons expressing the stimulatory DREADD hM3Dq in mice and rats produced increased neuronal excitability and pronounced decreased in sociability, without producing aversion, or changes in locomotor activity. This effect was specifically driven by PFC-LHb projections (Benekareddy et al., 2018).

LHb projections from the basal forebrain may also play a role in mediate social behaviors, specifically aggression (Golden et al., 2016). *In vivo* BF-LHb neuronal activity in aggressive versus non-aggressive CD-1 mice was significantly higher during a resident-intruder task. Neither, activation (AAV-hSYN-ChR2-eYFP), nor inhibition (AAV-hSyn-NpHR3-eYFP) of BF-LHb terminals altered attack latencies during the task. However, upon introduction of an intruder mouse into the home cage, stimulation of NpHR3 BF-LHb terminals in aggressive mice, attenuated the duration of attack. Conversely, stimulation of ChR2 BF-LHb terminals enhanced the duration of the attack (Golden et al., 2016). Stimulation of BF-LHb terminals was also shown to potentiate the rewarding effects of cocaine, but no modulation of anxiety-like behavior or social reward were detected following BF-LHb terminal photostimulation (Golden et al., 2016).

Together these interesting studies demonstrate the broad spectrum of social behaviors influenced by the LHb. Given the prevalence of social deficits across several neuropsychiatric disorders, it is clear the continued exploration of the LHb in the integration of social behaviors is warranted.

## MDD, Stress, and LHB Dysregulation

Perhaps the most compelling evidence implicating LHb dysregulation in MDD comes from deep brain stimulation (DBS) studies in humans. DBS of the LHb has the potential to produce rapid and sustained relief from depression for severely ill patients. In one female subject with chronic unremitted depression, bilateral LHb DBS surgery produced remission of depressive symptoms 4 months after surgery (Sartorius et al., 2010). Concurrent to DBS treatment, serum samples were collected to measure BDNF levels in this patient over the course of her treatment. Baseline levels of BDNF 9 to 3 weeks prior to surgery were 3.1 ± 1.7 ng/ml. Following surgery, levels increased to 5.8 ± 2.0 ng/ml, and by week 14 BDNF levels had risen to 8.2 ± 2.2 ng/ml (Hoyer et al., 2012). The rise in BDNF serum levels paralleled previous findings with electroconvulsive therapy (ECT) and antidepressant pharmacotherapy (Kishi et al., 2017). Additionally, habenular volumes are dramatically diminished in MDD and bipolar patients compared with healthy controls (Ranft et al., 2010). Overall, as the LHb is positioned to influence maladaptive reward processing and cognitive impairment, exploring the impact of stress and antidepressant treatment outcomes in this region may identify therapeutic targets for the treatment of MDD (Fakhoury, 2017).

### Stress and LHb Metabolic Activity

Stress is one of the central risk factors in the development of affective disorders such as depression and anxiety. Mounting preclinical evidence, primarily from rodent studies, supports the hypothesis that LHb activity is dramatically increased following stress.

Several different stress procedures have been shown to elevate 14C-2-deoxyglucose (2DG) metabolism in LHb of rats, including amphetamine withdrawal, which dramatically decreased catecholamine levels, α-methyl-para-tyrosine (AMPT) administration which inhibited brain norepinephrine and dopamine synthesis, and exposure to CMS (Caldecott-Hazard et al., 1988). These dramatic increases in metabolism occurred in parallel to reductions in locomotor and rearing behaviors in the open field test, reminiscent of a depressive-like phenotype in rodents (Caldecott-Hazard et al., 1988). Metabolic 2DG changes in the LHb and anxiety-like behavior were normalized following treatment with the antidepressant tranylcypromine (Caldecott-Hazard et al., 1988). Moreover, congenital learned helplessness (cLH) rats, which have increased susceptibility to LH and display exaggerated freezing response to shock, exhibited a dramatic increase in LHb CO metabolism (71%) that correlated with a 28%, 14%, and 16% reduction in metabolism in the VTA, basal ganglia and both BLA and CeA, respectively (Shumake et al., 2003). LH effects on LHb metabolism were also evident in a 2-deoxy-2[(18)F]fluoro-D-glucose (18)FDG, positron emission tomography (PET) study conducted in male Sprague-Dawley rats, where increased metabolic activity was observed in the LS and LHb (Mirrione et al., 2014). In contrast with the data indicating elevated metabolic activity following stress, 2 days of water deprivation decreased methionine incorporation into the LHb (Lepetit et al., 1988) suggesting that stressors can differentially modulate the activity of the LHb and that aversive stressors require greater metabolic demands on the LHb. In relation to LHb metabolic activity following antidepressant treatment, only evidence from acute administration is available in the literature. Selective serotonin reuptake inhibitors (SSRIs), fluoxetine (40 mg/kg), paroxetine (4 mg/kg), fluvoxamine (4 mg/kg) and sertraline (4 mg/kg) reduced the cerebral glucose metabolism in the LHb of awake Fischer-344 rats, indicating that LHb activity is decreased by antidepressants in healthy stress-naïve animals (Freo et al., 2008). Acute administration of the weak anxiolytic and antidepressant buspirone, an agonist of presynaptic 5-HT1A receptors and weak antagonist of dopamine D2 receptors, increased glucose metabolism in the LHb (Freo et al., 1995), suggesting divergent effects of 5-HT and DA inputs on LHb metabolic activity.

### LHb, Stress, and CRF

Exposure to a stressful stimulus activates the hypothalamic–pituitary–adrenal (HPA) axis facilitating the release of the stress hormone corticotropin releasing factor (CRF, also known as corticotropin releasing hormone, CRH) from the parvocellular cells of the PVN, which activate extrahypothalamic CRF receptors in diverse brain regions that influence behavioral responding. Dysregulation of this system has been strongly linked with the development of affective disorders (Bangasser and Valentino, 2014). In contrast with the MHb which does not express CRF receptor 1 (CRFR1) positive cells in mice, LHb CRF expression increases over the course of development from P4 onward (Rosinger et al., 2017), highlighting an important distinction between the habenular nuclei. Restraint stress in male Sprague-Dawley rats activated CRF positive neurons in the PVN to a similar extent when the stress occurred in the light or dark cycle, as measured by measured by increased expression of the immediate early gene c-Fos. In contrast, LHb CRF positive cells greatly increased activation when stress exposure occurred during the dark phase of the cycle (Chastrette et al., 1991). This effect was driven by a significant decrease (61%) in the number of overall c-Fos positive cells observed in the nighttime exposed vs. daytime exposed animals, highlighting the importance of circadian rhythmicity in the responsiveness of the LHb to stress. Similarly, elevated c-Fos immunoreactivity in the LHb in male Wistar albino rats administered i.c.v. CRF injection paralleled the pattern of c-Fos expression induced by exposure to acute foot shock and acute restraint (Imaki et al., 1993). Moreover, light enhanced startle (startling tone 90–105 Db, paired with a light stimulus of 900 lux), but not fear potentiated startle (tone paired with 0.6 mA foot shock) dramatically enhanced c-Fos immunoreactivity in the LHb (Veening et al., 2009). c-Fos was also preferentially elevated by light enhanced startle in the ACC, LS, NAc shell and the area postrema, all of which modulate LHb activity (Veening et al., 2009). These data indicate that the LHb maybe more highly activated in environments that evoke greater levels of anxiety.

Overall, the effects of acute stress can be mitigated by LHb lesions. Open field exploratory and locomotor activity of adult male Sprague-Dawley rats exposed to a series of footshocks was reduced relative to non-stressed controls, but bilateral electrolytic lesions of the LHb attenuated this anxiety-like response (Lee and Huang, 1988). Similarly, intra-LHb kainic acid injections facilitated LHb mediated inhibitory avoidance and anxiogenic behavior in the elevated T maze (Pobbe and Zangrossi, 2008). Moreover, the LHb was preferentially activated (c-Fos immunoreactivity) in C57BL/6J mice following chronic restraint stress rather than exercise (Kim and Han, 2016), emphasizing the responsivity of the LHb to aversive stimuli.

The developmental importance of the LHb in modulating stress responsivity was demonstrated following water immersion restraint stress where transgenic animals, in which the immediate early gene promotor zif268/egr1 was fluorescently visualized, exhibited enhanced maturation of the LHb response to stress at P10 when immunopositive cells were not detected in unstressed developing rats (Ichijo et al., 2015). Furthermore, acute exposure to escapable and inescapable shock increased c-Fos expression in LHb-DRN projecting neurons (Dolzani et al., 2016), supporting previous findings that demonstrated the importance of LHb activation in DRN stress coping strategies and the emergence of depressive-like behavior (Amat et al., 2001; Sachs et al., 2015). Failure to adapt to environments can lead to exaggerated HPA activity, as observed in rats in which excitatory LHb transmission was inhibited by blockade of glutamatergic α-amino-3-hydroxy-5-methyl-4-isoxazolepropionic acid (AMPA) and kainate receptors with 6-cyano-7-nitroquinoxaline-2,3-dione (CNQX). These rats exhibited memory deficits in the Morris water maze, which were accompanied by exaggerated plasma concentrations of the stress hormone corticosterone, reminiscent of the corticosterone concentrations that were measured in animals during the first exposure to the task (Mathis et al., 2018), which suggests that the stress response to aversive stimuli requires LHb engagement for adaptation to stress at a physiological level.

Coping strategies in the forced swim test (FST) were augmented by intra-LHb endocannabinoid ligands. The type-1 cannabinoid receptor (CB1R) agonist WIN 55,212-2 produced a prodepressive phenotype, or passive coping strategy as measured by increased immobility, in both male and female rats. In contrast, male and female rats treated with the CBR1 antagonist rimonabant exhibited enhanced climbing behaviors, active coping, compared to vehicle treated animals (Berger et al., 2018). Similarly, effects were demonstrated in tasks relevant to anxiety-like behavior including the elevated plus maze and novelty suppressed feeding, where rimonabant produced anxiolytic-like effects but WIN 55,212-2 was ineffective (Berger et al., 2018). Coping strategies in response to acute stress may be regulated by LHb projections to the RMTg. Enhanced LHb Ca^2+^ fluorescence, indicative of increased LHb neuronal activation, correlated with increased immobility in FST, and reductions in motivation during an appetitive PR task (Proulx et al., 2018). Specifically, excitation, or inhibition of LHb-RMTg terminals by light stimulation enhanced and blocked these deficits, respectively (Proulx et al., 2018).

These data collectively highlight the effects of acute transient stressors on the LHb CRF system and neuronal activation. Bidirectional modulation of motivation to exert effort is an important consideration in the context of depressive-like states. However, the most compelling evidence implicating stress evoked LHb activation has emerged from studies evaluating the effects of chronic stress exposure.

### Chronic Stress and Early Life Exposure Influences LHb Activity

Exposure to chronic stress procedures in adulthood such as CMS or chronic social defeat, produces significant behavioral deficits across a range of rodent tasks relevant to depression and anxiety including the FST, sucrose preference, social interaction, novelty suppressed feeding and other anxiety-like tests (Yang et al., 2008, 2014; Meng et al., 2011; Cui et al., 2014; Lim et al., 2015; Febbraro et al., 2017; Jacinto et al., 2017; Berger et al., 2018; Zhang H. et al., 2018; Zhang S. et al., 2018), which are attenuated by inactivation of the LHb. Specifically, LHb lesion, DBS or pharmacological lesions restore monoamine levels and facilitates recovery of behavior. Inactivation of the LHb following lidocaine injection attenuated stress-induced elevated LHb glutamate levels, restored DRN 5-HT levels, and reversed the depressive-like phenotype of adult rats exposed to neonatal clomipramine administration (Yang et al., 2008). Moreover, tryptophan hydroxylase 2 (R439H) knock-in (Tph2KI) mice, which display 60–80% reductions in brain 5-HT, exhibit increased sensitivity to the social interaction deficits produced by chronic social defeat exposure (Sachs et al., 2015). These mice are insensitive to the effects of chronic antidepressant treatment and exhibit increased activation of LHb. Silencing of the LHb through a stereotactic injection of AAV8-hSyn-M4D (Gi)DREADD-mCherry, was sufficient to reverse the social interaction deficits induced by chronic stress exposure (Sachs et al., 2015). High frequency bilateral stimulation of the LHb dramatically reversed anxiety-like and depressive-like behavior of rats exposed to CMS in the home cage emergence, open field, and FST, respectively (Lim et al., 2015). These data emphasize the importance of LHb-DRN projections in stress and treatment response.

Two distinct LHb projections to the DRN are known to exist, a glutamatergic population (Kalen et al., 1986), and a set of substance P immunoreactive neurons (Neckers et al., 1979). Microinjection of substance P directly into the LHb of adult male Wistar rats exposed to CMS for 21 days, and in animals exposed to neonatal clomipramine administration, produced antidepressant responses during the FST, which correlated with elevated 5-HT concentrations in the DRN (Yang et al., 2014). Furthermore, pathway analysis of a microarray conducted on LHb samples collected from CMS exposed escitalopram non-responder and responder rats, revealed that recovery of stress-induced behavioral deficits was dependent on the restoration of growth factor receptor signaling for VEGF and EGF, histamine H1 receptor signaling, oxytocin receptor mediated signaling, Wnt signaling, glutamine glutamate conversion and transcription regulation by bZIP (Christensen et al., 2013), which is typically required for transcription factors such as cAMP response element-binding protein (CREB) to initiate transcription of neurotropic factors and neuropeptides like CRF and endogenous opioids.

In line with these findings, early-life stress in the form of maternal deprivation (MD) increased LHb intrinsic excitability in adolescent rats, associated with significant reduction in medium afterhyperpolarization (mAHP) amplitude and higher input resistance (Authement et al., 2018). This study demonstrated that in non-MD animals, CRF increased the excitability of LHb neurons and depressed GABAergic transmission onto LHb neurons through CB1R signaling (Authement et al., 2018). It has also been shown the CMS exposure selectively increased 2-arachidonoylglycerol (2-AG)/CB1R signaling in the LHb, which in turn disrupts the necessary monoacylglycerol lipase activity in the LHb required for hydrolysis of 2-AG. This deficit has been proposed as the mechanism through which chronic stress enhances LHb burst firing and reduced CB1R availability (Berger et al., 2018), thus mediating the behavioral deficits associated with stress. Additionally, the CB1R agonist WIN 55,212-2 has been shown to produce hypometabolism in the LHb and disrupted recognition memory (Mouro et al., 2018). These data are counterintuitive as CB1 activation is thought to produce antidepressant activity following acute administration (Hillard and Liu, 2014), but these data support a more complex picture of endocannabinoid function in the LHb and support future investigation of endocannabinoid signaling in LHb monoaminergic transmission in the context of early life and chronic stress.

Postsynaptic CRFR1 mediates hyperexcitability in the LHb through Ca^2+^ dependent activation of potassium SK channels in a PKA-dependent manner. As MD increased PKA signaling within the LHb, the effects of CRF on LHb intrinsic excitably were blunted and blockade of PKA signaling restored LHb intrinsic excitability in MD rats (Authement et al., 2018). Previously, it had been determined that even a short exposure to inescapable footshock or restraint plus tail shock was sufficient to facilitate long-term potentiation of a specific subset of LHb neurons, which was associated with increased phosphorylation of CREB (Park et al., 2017b). This matches with earlier data obtained from a cLH model of depression, where an increase in the proportion of cells displaying high-frequency miniature excitatory postsynaptic currents (mEPSCs) in LH animals, increased from 2% to 14–20% in helpless animals (Li et al., 2011). DBS of the LHb restored escape behavior in helpless rats, which correlated with suppression of the enhanced excitatory synaptic activity onto VTA-projecting neurons from the LHb in helpless animals with or without stress exposure (Li et al., 2011). The use of DBS was also employed to attenuate the depressive phenotype and LHb hyperexcitability that emerged in adult rats with prior exposure to maternal separation from P7-15 for 6 h per day (Tchenio et al., 2017). Although the LH models promotes a depressive state that is potentiated by fast excitatory transmission onto LHb-VTA neurons, data from the early life stress studies (Tchenio et al., 2017; Shepard et al., 2018) suggest that the depressive state is mediated by perturbation of GABA inhibition of LHb neurons in later life, and implicate EPN-LHb inputs in this mechanism. Specifically, it is postulated that reduced GABA_B_-GIRK signaling mediates LHb neuronal hyperactivity in MS animals (Tchenio et al., 2017), a process mediated by increased activity of phosphatases including protein phosphatase 2A (PP2A). Moreover, the inhibition of PP2A produced antidepressant-like effects in an LH model of depression in rats (Lecca et al., 2016). In addition, the putative antidepressant ketamine has also been shown to alleviate the hyperexcitability induced by a range of rodent models of depression including CMS and cLH (Cui et al., 2018; Yang et al., 2018).

### Sex-Specific LHb Changes Following Stress

Emerging from the literature are sex-specific traits of metabolism, activation and responsivity to a variety of stressful stimuli. Females exhibited greater stress evoked neuronal activity of the LHb (c-fos activation) following acute immobilization stress compared to males (Sood et al., 2018). Sex differences in the metabolic activity of LHb have also been reported, where female rats exhibited higher metabolic activity of the LHb at baseline and following stress exposure, including restraint stress, or forelimb formalin injections (Brown et al., 1996). Shorter periods of stress have been shown to activate LHb in females more rapidly than in males (Hodes et al., 2015). The subchronic variable stress (6 days of three alternating stressors) specifically produce deficits in behavior that correlated with enhanced LHb suppression of VTA neurons exclusively, without altering the activity of VTA DA neurons and LC-VTA projecting neurons (Zhang S. et al., 2018), highlighting the importance of examining LHb function in both sexes in response to stressors of varying intensity and duration. Basal corticosterone concentrations in response to restraint stress in male and female Sprague-Dawley rats were augmented in a sex-specific manner by intra-LHb administration of the CB1R ligands (Berger et al., 2018). Intra-LHb administration of the agonist WIN 55,212-2 suppressed baseline and stress evoked corticosterone concentrations in males but not in females. Similarly, intra-LHb administration of the CB1R antagonist rimonabant enhanced corticosterone concentrations in males only (Berger et al., 2018). However, no sex differences in intra-LHb rimonabant’s antidepressant-like and anxiolytic-like effects were noted in the FST, elevated plus maze, or novelty suppressed feeding behavior (Berger et al., 2018). At a physiological level, chronic unpredictable stress exposure induced similar enhancement in firing rates of LHb neurons, although females had lower levels of neuronal firing at baseline and post stress when compared to males (Berger et al., 2018). However, male but not female rats exhibited an increase in the percentage of spikes occurring in burst. Additionally, non-stressed female rats had a significantly shorter burst interspike intervals (ISI) compared with male non-stressed rats, but following chronic stress exposure, female rats exhibited longer burst ISI compared to non-stressed females, this effect was not observed in male rats (Berger et al., 2018). Overall, these data demonstrate sexual dimorphism in LHb responsivity to stress and in the mechanism underlying the behavioral phenotypes evoked by stress. As such it is clear that further exploration of LHb sex-specific alterations are warranted and may ultimately help to discern the reasons why women are twice as likely as men to be diagnosed with a psychiatric illness (Kessler, 2003).

Taken together these data demonstrates a clear link between stress exposure and increased activation of the LHb. Additionally, a range of recent studies illustrate that different exposures to stress at different periods of sensitivity, ranging from neonatal stress exposure, to chronic and unpredictable stressors in adulthood, can induce hyperexcitability of the LHb through different mechanisms. Moreover, the finding that despite the differences in the duration of treatment required, novel and conventional antidepressant therapies reverse LHb hyperexcitability highlight the importance of the LHb in developing more selective and rapid acting therapeutic strategies for depression.

## Lhb Circuits

As aberrant processing of stimuli is central to the emergence of depressive state, the aversive/rewarding behavioral effects induced by optogenetic modulation of LHb inputs and outputs are reflected in Tables 1, 2, respectively.

**Table 1:** Afferent connections to the LHb in the reward pathway.

Afferent connection	Animal	Rhodopsin	Cell type	Behavior	Result	Reward/Aversion ↑↓	Reference
LPO-LHb	VGAT:Cre	ChR2	GABA	RTPP	Preference for light stimulation	↑	Barker et al., 2017
LPO-LHb	VGAT:Cre	ChR2	GABA	Shuttle task	Light seeking behavior	↑	Barker et al., 2017
LPO-LHb	VGAT:Cre	ChR2	GABA	CPP	No preference	←→	Barker et al., 2017
LPO-LHb	VGLUT2:Cre	ChR2	Glutamate	Shuttle task	Light escaping behavior	↓	Barker et al., 2017
LPO-LHb	VGLUT2:Cre	ChR2	Glutamate	RTPP	Aversion to light stimulation	↓	Barker et al., 2017
LPO-LHb	VGLUT2:Cre	ChR2	Glutamate	CPP	Aversion for light conditioned room	↓	Barker et al., 2017
LHA-LHb	VGLUT2:Cre	ChR2	Glutamate /GABA	RTPP	Aversion to light stimulation	↓	Stamatakis et al., 2016
LHA-LHb	VGLUT2:Cre	NpHR3	Glutamate /GABA	RTPP	Preference for light stimulation	↑	Stamatakis et al., 2016
LHA-LHb	VGLUT2:Cre	NpHR3	Glutamate /GABA	Free lick task	Increase consumption, number of licks, and decrease time between licks	↑	Stamatakis et al., 2016
EP-LHb	Sprague-Dawley	ChR2	Glutamate /GABA Glutamate	RTPP	Aversion to light stimulation	↓	Shabel et al., 2014
EP-LHb	Sprague-Dawley	ChR2	Glutamate /GABA Glutamate	Shuttle task	Light escaping behavior	↓	Shabel et al., 2014
VP-LHb	VGLUT2:Cre	ChR2	Glutamate	RTPP	Aversion to light stimulation	↓	Faget et al., 2018
VP-LHb	VGAT:Cre	ChR2	GABA	RTPP	No preference	←→	Faget et al., 2018
VP-LHb	VGLUT2:Cre	ChR2	Glutamate	RTPP	Aversion to light stimulation	↓	Tooley et al., 2018

*Reward and aversion are indicated by ↑ and ↓ arrows, respectively. RTPP, real time place preference; CPP, conditioned place preference.*

**Table 2:** Efferent connections to the LHb in the reward pathway.

Efferent connection	Animal	Rhodopsin	Cell type	Behavior	Result	Reward/Aversion ↑↓	Reference
LHb-RMTg	C57BL/6J	ChR2	Glutamate	RTPP	Aversion to light stimulation	↓	Lammel et al., 2012
LHb-RMTg	C57BL/6J	ChR2	Glutamate	CPP	Aversion for light conditioned room	↓	Lammel et al., 2012
LHb-RMTg	C57BL/6J	ChR2	Glutamate	Negative reinforcement	Increased nose pokes to terminate stimulation	↓	Stamatakis and Stuber, 2012
LHb-RMTg	C57BL/6J	ChR2	Glutamate	Positive reinforcement	Decrease in nose pokes for sucrose	↓	Stamatakis and Stuber, 2012
LHb-VTA	VGLUT2:Cre	ChR2	Glutamate	RTPP	Aversion for light stimulation	↓	Lammel et al., 2012
LHb-VTA	VGLUT2:Cre	ChR2	Glutamate	CPP	Aversion for light conditioned room blocked by D1 antagonist in mPFC	↓	Lammel et al., 2012
VTA-LHb	C57BL/6J	ChR2	Glutamate	CPP	Aversion for light conditioned room blocked by D1 antagonist in mPFC	↓	Stamatakis et al., 2013
VTA-LHb	TH-IRES-Cre	ChR2	GABA	RTPP	Preference for light stimulation mitigated by gabazine not D1/D2 antagonist	↑	Stamatakis et al., 2013

*Reward and aversion are indicated by ↑ and ↓ arrows, respectively. RTPP, real time place preference; CPP, conditioned place preference.*

### LHb and Aversion

LHb glutamatergic projections terminate in the posterior RMTg where they influence responses to aversive stimuli (Lammel et al., 2012). After exposure to unpredictable foot shocks, voltage-clamp recordings from mice show an increase in frequency of mEPSCs and glutamate release without changes in postsynaptic glutamate properties (Stamatakis and Stuber, 2012). When photostimulated, ChR2 expressing mice show real-time place avoidance and with escape tendencies, a conditioned-place aversion, a preference to terminate photostimulation by nose poke, and a disruption of positive reinforcement compared to vector controls (Stamatakis and Stuber, 2012). Collectively, these behavioral studies demonstrate that glutamatergic signaling from the LHb to the RMTg promotes location-specific passive avoidance, conditioned avoidance, negative reinforcement of responses, and serves as punishment even in presence of a positive reward (Stamatakis and Stuber, 2012). Furthermore, enhanced LHb-RMTg transmission is suggested to impart reduced motivation to exert effort in the context of a depressive states, elevated immobility and reduced motivation to perform appetitive tasks were reversed following photoinhibition of LHb-RMTg terminals (Proulx et al., 2018). Additionally, photostimulation of LHb-RMTg projections did not alter motoric activity but rather produced a state of negative valence whether the effort expended by animals was reduced during tasks (Proulx et al., 2018).

Although most glutamatergic outputs from the LHb synapse on the RMTg, the VTA also receives glutamatergic inputs (Lammel et al., 2012; Stamatakis and Stuber, 2012). Unlike the LHb which is primarily glutamatergic, the VTA is a heterogeneous structure composed of dopaminergic (∼65%), GABAergic (∼30%) and glutamatergic neurons (∼5%) (Tan et al., 2012). There appear to be two distinct populations of LHb-VTA glutamatergic projections. Using TH-IRES-GFP mice to visualize dopaminergic neurons, (Stamatakis and Stuber, 2012) showed glutamatergic projections to the posterior VTA that localized on primarily non-dopaminergic neurons. Dopaminergic projections in the posterior VTA are associated with reward related behaviors mediated through the lateral shell of the NAc, as an increase in dopamine in the NAc is rewarding (Lammel et al., 2012). When the LHb-VTA projections expressing ChR2 were stimulated ∼60% of the VTA-NAc lateral shell neurons showed inhibitory postsynaptic currents (IPSCs) (Lammel et al., 2012). Since this effect was blocked by picrotoxin, a GABA_A_ antagonist, these LHb-VTA projections likely synapse on GABAergic interneurons in the posterior VTA and cause inhibition of dopamine signaling to the NAc through feed-forward inhibition.

Another group of LHb-VTA neurons project to the medial VTA. These neurons co-localize with dopaminergic neurons that project primarily to the medial prefrontal cortex (mPFC). When the neurons were stimulated, the mPFC showed increases in c-Fos expression (Lammel et al., 2012). These results suggest that LHb-VTA neurons target dopaminergic neurons projecting to the mPFC. Unlike reward-related dopaminergic signaling to the NAc, dopaminergic signaling from the VTA to the mPFC is aversive (Lammel et al., 2012). Phasic photostimulation of LHb-VTA neurons expressing ChR2 produced condition place aversion in the open field test compared to controls. Interestingly, low-frequency stimulation did not induce aversion, nor did phasic stimulation have an effect of anxiety or locomotor activity (Lammel et al., 2012). Microinjection of a D_1_ antagonist into the mPFC, abolished conditioned place aversion after photostimulation suggesting that the location specific avoidance is dependent on dopaminergic signaling from the VTA to the mPFC (Lammel et al., 2012).

Projections from the VTA to the LHb also mediate reward related behaviors. VTA-LHb neurons have a complicated phenotypic picture: the majority of the mesohabenular neurons co-express VGluT2 and VGAT2, with ∼30% also expressing tyrosine hydroxylase (TH) (Root et al., 2014a). Ultrastructural analysis with immunoelectron microscopy revealed that VTA-LHb projections formed asymmetric, symmetric, and puncta adhaerentia synapses with the majority being symmetric and asymmetric (Root et al., 2014b). Light stimulation of mesohabenular brain slices from VGluT2-ChR2 mice revealed both inward and outward currents that were blocked by an AMPA and GABA_A_ antagonist respectfully. TTX blocked both currents suggesting that GABA and glutamate are released from a monosynaptic axon terminal (Root et al., 2014b).

Behaviorally, the release of GABA or glutamate play different roles in aversion/reward. Stimulation of VTA-LHb axonal projections expressing ChR2 caused real-time place avoidance and conditioned place aversion in VGluT2:Cre mice (conditional expression only in glutamatergic neurons) (Root et al., 2014a). When the LHb was injected with glutamate antagonists (NMDA and AMPA receptors), photostimulation-induced aversion behavior was mitigated indicating that glutamatergic signaling from the VTA-LHb is aversive. Alternatively, a small population of dopaminergic neurons use GABAergic signaling to activate a reward-related phenotype. When transgenic TH-IRES-Cre (conditional dopamine positive neuronal expression) mice expressed ChR2 in the mesohabenular neurons, photostimulation caused a real-time place preference for the chamber where they received photostimulation (Stamatakis et al., 2013). This preference was obliterated by gabazine, but not by a D1/D2 antagonist, suggesting that place preference was mediated by GABA and not dopamine. Furthermore, the same mice displayed an increase in nose pokes when paired with photostimulation. These data suggest that GABAergic firing from the VTA-LHb is not only rewarding but can also be reinforcing. The GABAergic activity presumably suppresses the LHb leading to disinhibition of the VTA dopamine neurons described earlier.

Although the details are not entirely clear, this dual release of GABA/glutamate could act as a feedback system. If the habenula is overactive, the VTA may use GABAergic signaling to dampen the effect and vice versa. However, how this mechanism of control of the mesohabenular firing operates is still unclear. Together these results help paint a picture of how LHb glutamatergic projections work to create aversive stimuli through three mechanisms: (1) inhibition of DAergic firing by stimulation of GABAergic neurons in the RMTg, (2) inhibition of DAergic firing by direct stimulation of GABAergic interneurons in the posterior VTA that project to the NAc lateral shell, and (3) direct synapse on to DAergic neurons in the VTA projecting to the mPFC. These results show that different populations of dopamine neurons within the VTA have distinct roles in reward/aversion.

Recent evidence also implicates specific VP-LHb projects in the phenotypic expression of aversion and negative affect (Knowland et al., 2017; Faget et al., 2018; Tooley et al., 2018). Traditionally, VP neurons were treated as a homogenous population of GABAergic projections to the LHb, VTA and thalamic nuclei, which were activated in response to cues predicting reward (Tindell et al., 2006; Young et al., 2014). However, emerging evidence suggest that the VP has considerable heterogeneity in cell type, morphology and function, which all influence behavior (Root et al., 2015; Knowland et al., 2017). Indeed, it has been proposed that distinct VP parvalbumin (PV) positive neuronal projections to the LHb and VTA mediate behavioral despair and social withdrawal, respectively (Knowland et al., 2017). In that study, approximately 83% of the PV positive VP terminals in the LHb were vGluT2 positive and 19% were VGAT positive (Knowland et al., 2017). Although the majority of VP neurons are GABAergic, a recent study utilized DIO-mCherry injection into the VP of vGluT2-IRES-cre mice to visualize glutamatergic projections and determined that PV was co-expressed in 17% of these neurons, and ChAT was co-expressed in 4.3% of these neurons, highlighting potential heterogeneity in VP neurons (Tooley et al., 2018). Following this the authors conducted a series of anterograde and retrograde labeling to establish synaptic connectivity between these VP glutamatergic neurons and the LHb, and demonstrated increased firing rates in LHb neurons simulated by glutamatergic VP neurons (Tooley et al., 2018). Behaviorally, non-selective stimulation of the VP neurons produced conditioned place preference. However, selective stimulation of glutamatergic VP neurons at 20 Hz produced robust conditioned place avoidance (Tooley et al., 2018). Similarly, in a progressive ratio task where mice are trained to respond for sucrose pellets, ablation of glutamatergic VP neurons using AAV2-flex-taCasp3-TEVp dramatically increased the number of lever presses and increased the breakpoint in mice in this task. Additionally, in a sucrose aversion task, taCasp lesion mice continued to exhibited sucrose consumption despite an aversive injection of lithium chloride relative to control animals (Tooley et al., 2018). In another recent study, these glutamatergic neurons were localized to the ventromedial portion of the rostral VP (Faget et al., 2018). This study explored the reinforcing value of GABA and glutamatergic VP-LHb projections. A clear demonstration of VP-LHb GABA efferents promoting reinforcement of reward was evident in an intracranial self-stimulation task, where mice with ChR2:YFP expressing PV positive VP neurons exhibited a robust preference for nosepoke hole associated with the highest frequency stimulation (40 Hz) following light stimulation in the LHb. Furthermore, increased self-stimulation of mice expressing ChR2:YFP PV positive VP neurons was also associated with increased activity in VTA neurons (Faget et al., 2018). In contrast, light stimulation of LHb in mice expression ChR2 in VGluT2 positive VP neurons failed to self-stimulate, but it did induce robust real time place avoidance (Faget et al., 2018) Together these data confirm the importance of this specific subset of glutamatergic VP-LHb neurons in constraining reward seeking behavior in the presence of aversive cues and the robust induction of aversion (Faget et al., 2018; Tooley et al., 2018).

### LPO-LHb and Reward

The hypothalamic LPO influences LHb control of aversion and reward. The hypothalamus in general has been shown to modulate learning, emotion, reward, escape, and aggression behaviors (Ono and Nakamura, 1985). Lesion studies of the LPO show disruption in glucose preference suggesting a possible role in reward preference (Mook et al., 1975). Further, afferents from the LPO to the LHb have been shown to utilize GABAergic transmission. An increase in GABA tone would suggest that inputs from the LPO would be rewarding. However, recent optogenetic studies suggest a more complicated model for the LPO-LHb connection. The LPO-LHb sends glutamatergic axonal projections that have terminals on the dendrites (Barker et al., 2017). The global distribution of glutamatergic axonal terminals within the LHb shows an asymmetric distribution favoring termination in the medial LHb. LPO-LHb GABAergic projections showed a broad distribution throughout both the lateral and medial potions of the LHb and synapse both on dendrites and cell bodies (Barker et al., 2017). Surprisingly, the majority of LHb neurons receive both glutamatergic and GABAergic neurotransmission. In contrast to earlier hypothesis, these data suggest that LPO-LHb requires both GABA and glutamatergic input.

Behaviorally, stimulation of these neurons showed a distinct phenotype based on the population of neurons stimulated (Barker et al., 2017). Glutamatergic afferent LPO-LHb stimulation caused photostimulation induced place aversion, in which mice spent less time in the chamber with photostimulation and exhibited aversion to that chamber when no photostimulation was present. Furthermore, during a shuttle avoidance task, photostimulation of glutamatergic inputs evoked escape behaviors. GABAergic stimulation produced opposite results as the mice spent more time in the photostimulation chamber, however, they did not develop a preference for the chamber during non-stimulation trials. Also, photostimulation of these GABAergic neurons produced a positive response in the shuttle reward task. Interestingly, during footshock, both GABAergic and glutamatergic inputs release Ca^2+,^ but not during consumption of sucrose nor in response to a cue predicting its delivery (Barker et al., 2017). Together this information gives a better understanding of how this dual signaling may play a role in noxious stimuli processing. This gives a bivariate control of input and these results suggest that a balance of GABAergic and glutamatergic release from the LPO afferents is necessary for normal reward and aversion processing.

### LHA-LHb and Feeding

An interesting area of reward-related connection to the LHb is the LHA, which is implicated in feeding and reinforcement processes (Jennings et al., 2013). Motivation for food-based rewards is key for survival as appropriate consumption of high caloric meals is required to sustain metabolic needs (Jennings et al., 2013). In a survival situation, the need to consume calories can often overrule normal incentive salience. The LHA is a heterogeneous structure containing neurons releasing GABA, glutamate, and the neuropeptides melanin-concentrating hormone, and orexin (Jennings et al., 2015) with projections to the thalamus, cortex, midbrain, and hindbrain (Stamatakis et al., 2016). Electrical stimulation of the LHA leads to feeding and reward-base behaviors (Jennings et al., 2015). Stimulation of the LHA with ChR2 in VGAT-IRES-Cre mice led to increases in time spent in food-related area and food consumption and to nose poked induced self-stimulation. Inhibition of these GABAergic neurons with NpHR3 produced the opposite effect (Jennings et al., 2015). These results confirm that GABAergic outputs from the LHA are both rewarding and lead to increased feeding. Not surprisingly, LHA-LHb are both GABAergic and glutamatergic. LHA-LHb glutamatergic projections are found in the LHA with the highest density in the anterior portion of the LHA. Voltage clamp recordings of VGluT2-IRES-Cre mice expressing ChR2 in the LHA-LHb neurons showed that the main effect of this glutamatergic input was to increase the firing rate of postsynaptic neurons. However, the presence of both EPSC and IPSC that are attenuated by DNQX and gabazine respectfully suggest the release of both glutamate and GABA (Stamatakis et al., 2016). Although there is a clear postsynaptic GABA component upon stimulation of VGluT2 LHA-LHb neurons, the mechanism by which GABA is released remains unknown, since glutamatergic and GABAergic neurons within the LHA have been shown to be distinct populations (Sharpe et al., 2017). Behaviorally, photostimulation of ChR2 caused a real-time place aversion while photostimulation of the same mice expressing NpHR3 caused real-time place preference. In these same mice, NpHR3 photostimulation during a free licking task increased consumption of a highly palatable calorically dense liquid, number of licks, and decreased the amount of time in-between licks. These results suggest that LHA helps control food related reward-based decisions through the amount of activation of the LHb.

The LHA also exerts control over reward through its GABAergic outputs to the VTA. However, the population of neurons that are activated are possibly input specific. One possible key input that may influence LHA-LHb activation is the bed nucleus of the stria terminals (BNST). The BNST sends GABAergic inputs to the LHA and VTA. Photostimulation of ChR2 of BNST-LHA projections in VGAT-IRES-Cre mice exhibited a real-time place preference as well as an increase in photostimulation paired nose pokes (Jennings et al., 2015). Even in well fed mice, activation of LHA increased consumption of food with high fat consumption increasing the most. On the other hand, inhibition of this circuit via NpHR3 led to a reduction of these consummatory and reward-related behaviors even when food restricted. This behavior was specific to BNST-LHA circuitry, and did not extend to BNST-VTA connections, suggesting the GABAergic BNST output is LHA specific. More importantly, BNST-LHA projections synapse on to glutamatergic neurons in the LHA. Although studies have not confirmed this connection directly, activation of the BNST could theoretically lead to the inhibition of LHA-LHb projections.

### EPN-LHb, Behavioral Despair, and Goal Directed Performance

Another major connection of the reward system to the LHb arises from the basal ganglia. Although classically associated with motor control, the basal ganglia is implicated in associative and limbic functions (Alexander et al., 1986). One of the major output areas of the basal ganglia is the GPi. Clinically, patients with a stroke in the GPi have presented with anhedonic symptoms (Howell et al., 2016). Furthermore, during DBS in an awake human subject, single unit recordings of non-motor neurons during a reward task changed firing rate based on reward value (Howell et al., 2016). Non-human primates have shown comparable results as activation of GPi increased goal-oriented actions in the presence of reward information (Howell et al., 2016). Studies in rodents have shown similar non-motor functions of select neurons arising from the EPN projecting to the LHb, thalamus, and brainstem. The LHb receives input from the EPN via two distinct axonal projections. Most of the input from the anterior region of the EPN is from cells that express somatostatin and innervate the lateral potion of the LHb bilaterally. These somatostatin positive cells co-release glutamate and GABA (Wallace et al., 2017). Another group of neurons from the posterior portion of the EP that express parvalbumin (PV) project to the oval nucleus of the LHb and are a source of ipsilateral glutamatergic input (Wallace et al., 2017).

When VGluT2:Cre mice expressing ChR2 in EPN-LHb projections received a 50 Hz stimulation in the presence of picrotoxin, the firing rate increased compared to non-picrotoxin stimulation. This same increase, however, was not seen with 2 Hz stimulation. This suggest the role of GABA and glutamate co-release may be to dampen the effect of glutamate hyperactivity (Shabel et al., 2014). This reduced GABA/glutamate ratio induced by various rodent models of depression can be reversed by antidepressant treatment (Shabel et al., 2014). This LHb input is necessary for the evaluation of action outcome or determining whether the outcomes are better or worse than expected. ChR2 expression in EPN afferents produced real-time place avoidance compared to YFP controls when photostimulation occurred in the LHb (Shabel et al., 2014). When EPN-LHb axons from the lateral portion of the LHb were voltage-clamped, application of serotonin caused presynaptic inhibition of light-induced excitability. These data suggest that the EPN-LHb pathway may be involved in reinforcement learning and encode negative reward prediction errors (Shabel et al., 2014).

### LHb Inputs and Depressive Behavior

In addition to EPN-LHb circuits, emerging evidence from optogenetic studies highlight the importance of the DRN-LHb circuit in restoring normal behavior following exposure to chronic stress. Optic suppression (AAV9-hSyn-eArch3.0-EYFP) of DRN-LHb projections elevated c-Fos expression in the LHb, producing significant reductions in sucrose preference but no change of performance in the FST (Zhang H. et al., 2018). The reduction of sucrose preference was prevented by intra-LHb application of 5-HT immediately prior to optogenetic silencing of DRN-LHb projections. Similarly, photostimulation of ChR2 expressing DRN-LHb terminals (AAV-hSyn-ChR2-mCherry) presynaptically inhibited the excitability of LHb neurons of rats exposed to CMS leading to increased levels of DRN 5-HT and reversing deficits of sucrose drinking (Zhang H. et al., 2018). Moreover, less than 10% of DRN inputs to the LHb were GABAergic; the remainder were inhibitory 5-HT terminals, the effects of which were abolished by intra-LHb 5-HT_1B_ receptor antagonist administration (Zhang H. et al., 2018).

Behavioral despair induced by LHb hyperactivity can be induced by PV positive VP-LHb projections. Marked increases in VP PV neuronal activity is evident following exposure to chronic social defeat, these neurons project long-range sending terminals to the LHb and the lateral VTA (Knowland et al., 2017). Light stimulation of VP PV projections exclusively inhibited GABAergic VTA neurons, conversely stimulation of VP PV terminals in the LHb evoked excitatory EPSCs in nearly all LHb neurons (Knowland et al., 2017). Moreover, stimulation of PV VP-LHb neurons drove conditioned place aversion, which was abolished by silencing these neurons, agreeing with previous findings that demonstrated the importance of LHb activity in mediating behavioral aversion (Shabel et al., 2012). The stress-induced hyperexcitability of these PV VP-LHb projections was attenuated by chronic, but not acute, administration of fluoxetine. Moreover, mice that were resilient to the behavioral effects of social defeat did not exhibit hyperexcitability in PV VP-LHb neurons, emphasizing the importance of this pathway in determining individual differences in stress susceptibility (Knowland et al., 2017).

Another potential candidate population of LHb neurons that drive depressive like behaviors are p11 positive neurons. As a multifunctional protein interacting with 5-HT receptors, ion channels, and other factors, p11 influences the response to antidepressant drugs and prodepressive responses in rodent models of depression (Svenningsson et al., 2013) Silencing this neuronal population by photostimulation of the LHb reversed social interaction deficits, depressive-like behavior in the FST, TST and novelty suppressed feeding that were produced by chronic restraint stress (Seo et al., 2018). At a physiological level, the burst firing of mice with LHb p11 knockdown was decreased relative to chronically stressed animals, but not abolished entirely (Seo et al., 2018). However, overexpression of p11 in D2R-containing glutamatergic LHb neurons was sufficient to induce depressive-like behavior. Approximately 88% of p11^+^/D2^+^ neurons expressed Ca^2+^/calmodulin dependent kinase II (CaMKII), with the remainder expressing vesicular GABA transporter.

**FIGURE 2:**
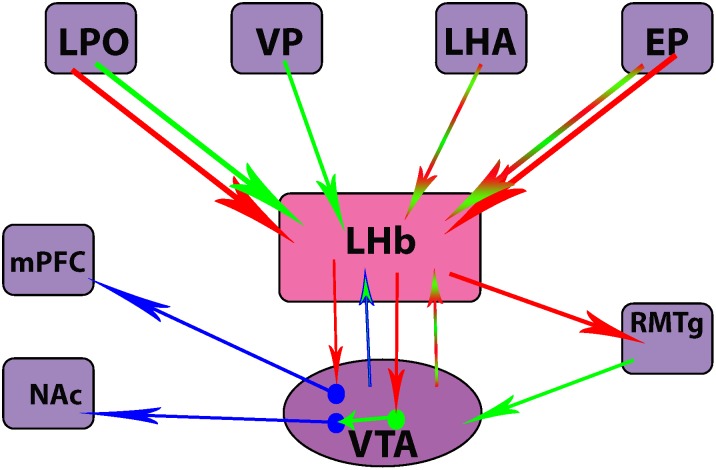
Differences in neurotransmitter release from efferent and afferent projections of the lateral habenula (LHb) nucleus. Clockwise from top left corner: lateral preoptic area (LPO), ventral pallidum (VP), lateral hypothalamic area (LHA), entopeduncular nucleus (EP), rostromedial tegmental nucleus (RMTg), ventral tegmental area (VTA), nucleus accumbens (NAc), medial prefrontal cortex (mPFC). Red = glutamatergic, green = GABAergic, blue = dopaminergic.

Overall, these optogenetic studies have yielded compelling information regarding specific populations of neurons in the LHb that are implicated in the emergence of depressive behaviors following stress. Additionally, these studies have highlighted the complex profile of neurotransmitter release from both efferents and afferents of the LHb (Figure 2). They may also represent potential therapeutic targets for more rapid and sustained relief from depressive symptoms.

## Molecular Mechanisms Underpinning Lhb Hyperactivity in Depression

### DNA Methylation

Transcriptional regulators of synapse formation are altered by stress. One such regulator, methyl-CpG-binding protein-2 (MeCP2), is stimulated by synaptic activity (Zhou et al., 2006), monoamine neurotransmitters (Deng et al., 2010) and by antidepressant treatment (Hutchinson et al., 2012). KI mice that carry a mutation eliminating the phosphorylation of MeCP2 at Ser42 exhibited increased sensitivity to stress. Unlike wild-type controls, these MeCP2 KI mice were resistant to the antidepressant effects of chronic imipramine given following exposure to social defeat stress (Hutchinson et al., 2012). In wild-type, but not MeCP2 KI mice, chronic imipramine treatment induced phosphorylation of pMeCP2 in a specific population of neurons located on the medial side of the LHb. This pattern of findings suggested that the ability of chronic imipramine to restore social interaction following exposure to social defeat stress is facilitated in part by DNA-methylation dependent synaptic restructuring of LHb neurons (Hutchinson et al., 2012).

### Increased Bursting Rate of Neurons

A potential molecular mechanism for LHb mediated depressive like behavior is the upregulation of T-type Ca^2+^ channels (T-VSCC) and subsequent initiation of NMDA-dependent neuronal bursting. In a depressive state, the pathological condition of low K^+^ concentrations facilitated the repeated activation of T-VSCC, facilitating the enhanced bursting pattern observed in the LHb during stress. Such low K^+^ concentrations may be more prevalent in a depressive like state, where the buffering of extracellular K^+^ is upregulated by increased activity of astroglial Kir4.1 potassium channels. Indeed, it was demonstrated by Cui et al. (2018) that knockdown of the astroglial but not the neuronal Kir4.1 gene within the LHb reversed the depressive like phenotype of rats selectively bred for cLH in response to inescapable shock. Supporting this hypothesis, the authors also demonstrated that the activity of astrocytic Kir4.1 channels was greatly enhanced following administration of lipopolysaccharide, an endotoxin which is commonly used to induce transient signs of depressive-like behavior (Cui et al., 2018). Moreover, a gain of function of Kir4.1, using viral infection (AAV-GFAP:Kir4.1) of the astrocytes in the LHb bilaterally in mice, produced greater hyperpolarization of astrocytes and glia, elevated the percentage of bursting neurons and robustly enhanced immobility levels in the FST and significant reductions in sucrose drinking 21 days post administration. Notably the enhanced activity of Kir4.1 channels was evident in rats age p60-90, but differences in Kir4.1 activity and current were not obvious in younger rats (P60), suggesting that there may be a crucial developmental period during which the LHb is most sensitive. In agreement with these observations, inhibitory opsins eNpHR3 drive burst firing in LHb and produced depressive like behaviors (Yang et al., 2018). In relation to antidepressant responses, DBS (Jacinto et al., 2017) and the putative antidepressant ketamine (Yang et al., 2018) reversed burst firing in LHb neurons.

As observed in human imaging studies, CMS exposure in male Wistar-Han rats produced atrophy of the LHb, which was attributed to a reduction in glial cells (Jacinto et al., 2017). This finding supports the hypothesis of Cui et al. (2014, 2018) that reductions in the capacity of glia to buffer extracellular glutamate facilitates the emergence of neuronal hyperexcitability in the LHb and depressive-like behavior. Indeed, inhibition of glial glutamate transporter GLT-1 increased the neuronal firing rate and c-Fos expression in the LHb, consequently increasing depressive like behaviors in the tail suspension test (TST) and novelty suppressed feeding test (Cui et al., 2014).

### GABA-GIRK Signaling

Perturbation of GABA inhibition of LHb neurons has emerged as a potential molecular mediator of LHb hyperexcitability and antidepressant treatment response (Shabel et al., 2014; Lecca et al., 2016; Tchenio et al., 2017; Authement et al., 2018). It has been postulated that GABA_B_-GIRK plasticity in the LHb is a cellular substrate for aversive experience (Lecca et al., 2016). Specifically, stress induced PP2A activity, mediated the internalization of GABA_B_ receptors and G protein-gated inwardly rectifying potassium (GIRK) channels (Lecca et al., 2016), which subsequently diminished GABA_B_-activated GIRK-mediated currents. Additionally, PP2A activity was increased in activated P11 positive LHb neurons, which positively correlated with depressive like behaviors (Seo et al., 2018). Pharmacological inhibition of PP2A restored GABA_B_-GIRK function, neuronal excitability, and depression-like symptoms in a learned-helplessness model of depression. Furthermore, systemic administration of DA and glucocorticoid receptor antagonists was sufficient to block stress induced reductions in GABA_B_-GIRK currents (Lecca et al., 2017). However, the effects of these pharmacological manipulations were not exerted at the level of the LHb but most likely produced their effects by modulated other stress-responsive structures in circuits that project to the LHb.

### βCaMKII Signaling

Recent studies have shown that the increased depolarization of LHb neurons is potentially mediated through elevated βCaMKII function. Initially identified in a proteomic screen of LHb tissues following stress, overexpression of βCaMKII, but not αCaMKII, in the LHb was shown to induce depressive behaviors and anhedonia in rats (Li et al., 2013). Moreover, down-regulation of βCaMKII levels, blocking its activity or its target molecule the glutamate receptor GluR1 in cLH rats reversed depressive symptoms (Li et al., 2013). In contrast with the persistent lifelong stress disposition of cLH rats, acute stress increased both isoforms, αCaMKII and βCaMKII (Park et al., 2017a). Furthermore, CaMKII is implicated in producing the aberrant endocannabinoid signaling profile that facilitates LHb hyperexcitability following stress (Park et al., 2017a; Authement et al., 2018). CaMKII regulates long-term potentiation and multiple signal transduction pathways involved in learning and memory processes. Specifically, it is also known to facilitate the trafficking of AMPA receptors to increase postsynaptic responses to presynaptic depolarization and increases channel conductance of AMPA GluA1 subunits. Stress-induced elevations in βCaMKII signaling may relate to depression by influencing the formation and retention of aversive memories. DBS of the LHb reversed behavioral deficits in rats that were resistant to antidepressant treatment, and these behavioral changes were accompanied by reductions in the phosphorylation of αCaMKII and βCaMKII, GSK3α and GSK3β and AMPK in the LHb and infralimbic cortex (Kim et al., 2016). Increased βCaMKII expression was also detected in glutamatergic LHb neurons (Seo et al., 2018).

Overall these data illustrate a complex orchestration of molecular mediators in the emergence of LHb hyperexcitability following exposure to stress, in particular alterations in glutamate-GABA_B_ receptor cross-talk. Presynaptically, GABA_B_ heteroreceptors inhibit the release of glutamate, indirectly by reducing calcium influx and directly by inhibiting neurotransmitter release at the level of the SNARE complex. GABA_B_ autoreceptors on GABAergic terminals are also critical for regulating synaptic GABA concentrations. Postsynaptic GABA_B_ receptors located on the dendritic shaft and spine, are trafficked to and from the cell surface in a CaMKII dependent manner. These postsynaptic GABA_B_ receptors are activated by GABA spillover from local GABAergic terminals, which in turn induces slow IPSCs in a GIRK dependent manner (Luscher et al., 1997), ultimately hyperpolarizing the membrane and attenuating NMDA mediated excitatory currents. In a depressive state, GABA_B_ expression and function is diminished, promoting hyperexcitability. Conversely conventional antidepressants, electro convulsant shock and other antidepressant therapies restore GABA_B_ expression and function across many brain regions that influence the emergence and treatment of MDD (Jacobson et al., 2018). Thus, normalization of GABA/glutamate ratio, GABA_B_-GIRK currents and βCaMKII signaling may serve as potential molecular biomarkers of successful antidepressant response.

## Conclusion

Although the studies covered in this review provide evidence in support of the LHb hyperexcitability in the development and remediation of depression, more work is needed to consolidate this hypothesis. Additional LHb circuits that have been implicated in depression have not yet been evaluated. Fortunately, advances in pharmacological tools and optogenetics are making this type of mapping easier. In addition, the use of Designer Receptor Exclusively Activated by Designer Drug (DREADD) technology (Nair et al., 2013) has produced some exciting information pertaining to LHb circuits relevant to depression, aversion, motivation and stress reactivity. Already these tools have helped to accumulate evidence pertaining to the role of the LHb in control of midbrain release of monoamine neurotransmitters and their role in reward and aversive behaviors. In relation to the emergence of depression, one of the most interesting facets uncovered by these experiments is the dual GABA and glutamate inputs by all optogenetically studied nuclei projecting to the LHb. GABA tone in the LHb seems to be necessary to dampen glutamatergic spiking in response to aversive stimuli. This network can be advantageous as even the most aversive and rewarding stimuli profoundly impact cognition and motivation to respond for rewards which are significantly impaired in depression. It is also likely that different modulating burst firing patterns induced by multiple molecular mediators may provide new sophisticated tools that hold the key to alleviating LHb dysfunction in depression. Moreover, the striking common effects of acute administration of ketamine, endocannabinoids and chronic administration of conventional antidepressant have highlighted key molecular, neurophysiological and neurochemical targets that may serve as potential therapeutic targets for the rapid and sustained treatment of MDD and other stress-related disorders.

## Author Contributions

CB, RH, and IL wrote the article. CB and RH prepared the figures and tables.

## Conflict of Interest Statement

The authors declare that the research was conducted in the absence of any commercial or financial relationships that could be construed as a potential conflict of interest.
